# Safety and tolerability of once-daily umeclidinium/vilanterol 125/25 mcg and umeclidinium 125 mcg in patients with chronic obstructive pulmonary disease: results from a 52-week, randomized, double-blind, placebo-controlled study

**DOI:** 10.1186/1465-9921-15-78

**Published:** 2014-07-11

**Authors:** James F Donohue, Dennis Niewoehner, Jean Brooks, Dianne O’Dell, Alison Church

**Affiliations:** 1 Department of Medicine, University of North Carolina, Chapel Hill, NC, USA; 2 VA Medical Center, Minneapolis, MN, USA; 3 GlaxoSmithKline, Respiratory Medicines Development Centre, Stockley Park, Uxbridge, UK; 4 Research & Development, GlaxoSmithKline, Research Triangle Park, Durham, NC, USA

**Keywords:** Bronchodilator, Long-acting muscarinic antagonist, Long-acting β_2_-adrenergic agonist, Combination

## Abstract

**Background:**

The long-acting muscarinic antagonist (LAMA) umeclidinium (UMEC) and the combination of UMEC with the long-acting β_2_-agonist (LABA) vilanterol (UMEC/VI) are approved maintenance treatments for chronic obstructive pulmonary disease (COPD) in the US and EU. They are not indicated for the treatment of asthma.

**Methods:**

In this 52-week, double-blind, placebo-controlled, parallel-group safety study (GSK study DB2113359; NCT01316887), patients were randomized 2:2:1 to UMEC/VI 125/25 mcg, UMEC 125 mcg, or placebo. Study endpoints included adverse events (AEs), clinical chemistry and hematology parameters, vital signs, 12-lead, and 24-hour Holter electrocardiograms. COPD exacerbations and rescue medication use were assessed as safety parameters; lung function was also evaluated.

**Results:**

The incidence of on-treatment AEs, serious AEs (SAEs), and drug-related AEs was similar between treatment groups (AEs: 52–58%; SAEs: 6–7%; drug-related AEs: 12–13%). Headache was the most common AE in each treatment group (8–11%). AEs associated with the LAMA and LABA pharmacologic classes occurred at a low incidence across treatment groups. No clinically meaningful effects on vital signs or laboratory assessments were reported for active treatments *versus* placebo. The incidences of atrial arrhythmias with UMEC/VI 125/25 mcg were similar to placebo; for UMEC 125 mcg, the incidences of ectopic supraventricular beats, sustained supraventricular tachycardia, and ectopic supraventricular rhythm were ≥2% greater than placebo. With active treatments, COPD exacerbations were fewer (13–15% of patients reporting ≥1 exacerbation) and on average less rescue medication was required (1.6–2.2 puffs/day) *versus* placebo (24% reporting ≥1 exacerbation, 2.6 puffs/day). Both active treatments improved lung function *versus* placebo.

**Conclusion:**

UMEC/VI 125/25 mcg and UMEC 125 mcg were well tolerated over 12 months in patients with COPD.

## Background

Chronic obstructive pulmonary disease (COPD) is a preventable and treatable condition characterized by persistent airflow obstruction that is not fully reversible [1]. The pharmacological management of stable COPD primarily aims to improve symptoms and quality of life, optimize lung function, reduce COPD exacerbations, and improve exercise tolerance [1,2]. Bronchodilators are central to the pharmacological management of COPD, and include long-acting muscarinic antagonists (LAMAs) and long-acting β_2_-adrenergic agonists (LABAs). Muscarinic antagonists bind to M_3_ receptors, thereby blocking the bronchoconstrictive response to cholinergic nervous stimulation [3], while β_2_-agonists stimulate β_2_-adrenergic receptors and increase levels of cyclic adenosine monophosphate [2]. Both mechanisms facilitate airway smooth muscle relaxation.

The combination of these distinct and complementary mechanisms of action may provide the opportunity for improved treatment efficacy. Indeed, the co-administration of LAMAs and LABAs has been shown to produce significantly greater improvements in lung function compared with the monotherapy components in patients with COPD, as have their short-acting counterparts [4-7]. In addition to stabilizing lung function over 24 hours, the development of a LAMA/LABA combination treatment may also improve treatment adherence due to the convenience of a once-daily treatment regimen [8], and administration of both drugs via a single inhaler. A LAMA/LABA therapy may also be associated with a lower risk of side effects in comparison with increasing the dose of a single agent [2].

The LAMA umeclidinium (UMEC) and the combination of UMEC with the LABA vilanterol (UMEC/VI) are approved maintenance treatments for COPD in the US and EU. They are not indicated for the treatment of asthma. Previous studies have shown that both UMEC and VI can significantly improve lung function over 24 hours [9,10]. Studies have also demonstrated that UMEC and VI are well tolerated over a 6-month period [11,12], but data on longer-term exposure are lacking at present. This study was conducted to examine the safety and tolerability of once-daily UMEC/VI 125/25 mcg and UMEC 125 mcg compared with placebo over 12 months in patients with COPD.

## Methods

### Study design

This was a Phase IIIa, multicenter, randomized, double-blind, placebo-controlled, parallel-group study (GSK study number DB2113359; ClinicalTrials.gov identifier NCT01316887) conducted between January 2011 and July 2012. Patients who met eligibility criteria entered a run-in period of 7–10 days, followed by a 52-week treatment period. Patients who experienced a COPD exacerbation or lower respiratory tract infection (LRTI) during the run-in period or at Visit 2 were allowed to re-screen and repeat the run-in period.

The study was conducted in accordance with the International Conference on Harmonisation of Technical Requirements for Registration of Pharmaceuticals for Human Use Good Clinical Practice (ICH) guidelines, all applicable subject privacy requirements, and the ethical principles outlined in the Declaration of Helsinki, 2008 [13,14].

### Patients

Eligible patients were current or former smokers of ≥40 years of age, with a smoking history of ≥10 pack-years and an established clinical history of COPD as defined by the American Thoracic Society/European Respiratory Society criteria [1]. Patients had a post-salbutamol forced expiratory volume in one second (FEV_1_)/forced vital capacity (FVC) ratio <0.70 and a post-salbutamol FEV_1_ ≥35% and ≤80% of predicted values (as determined by Nutrition Health and Examination Survey III reference equations) [15]. Female patients were eligible for participation if they were of non-childbearing potential, or agreed to practice acceptable methods of birth control, as defined by the protocol.

Patients with a current diagnosis of asthma or other respiratory disorder (including pulmonary hypertension and interstitial lung disease) were excluded, as were patients with historical/current evidence of clinically significant, uncontrolled, cardiovascular, neurological, psychiatric, renal, hepatic, immunological, endocrine, or hematological abnormalities that the investigator felt may have put the patient at risk or affected the safety analysis of the study. Patients were also excluded if they: had been hospitalized for COPD/pneumonia within 12 weeks prior to Visit 1 or had undergone lung resection in the 12 months prior to screening; were hypersensitive to any anticholinergic drug or β_2_-agonist; were unable to withhold salbutamol and/or ipratropium bromide use for the 4-hour period prior to spirometry; had a known or suspected history of alcohol or drug abuse; were participating in the acute phase of a pulmonary rehabilitation program; or had abnormal and significant findings from electrocardiogram (ECG) monitoring, 24-hour Holter monitoring, chest X-rays, clinical chemistry, or hematology tests. Prohibited medications prior to study entry are summarized in Additional file 1: Table S1.

### Study treatments and randomization

**Figure 1:**
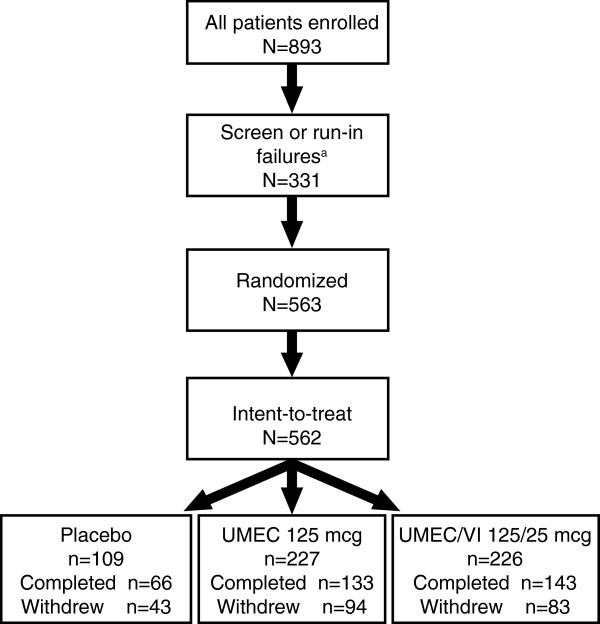
**Study design. **^a^One patient was randomized in error but did not receive study drug. UMEC, umeclidinium; VI, vilanterol.

Patients were randomized in a 2:2:1 ratio to once-daily UMEC/VI 125/25 mcg (delivering 113/22 mcg), UMEC 125 mcg (delivering 113 mcg), and placebo (Figure 1), using a telephone-based randomization system and codes generated by RandAll version 2.5. All treatments were administered in the morning via the ELLIPTA™ dry powder inhaler. Salbutamol and/or ipratropium bromide were permitted as rescue medication throughout the run-in and treatment periods, administered via metered dose inhaler or nebules.

### Outcomes and assessments

Safety assessments included the incidence of adverse events (AEs), vital signs and clinical chemistry, hematology, 12-lead ECG, and 24-hour Holter ECG parameters. AE groups of special interest, such as those associated with LAMA and LABA pharmacologic classes, were also assessed. These included: cardiovascular effects, effects on glucose and potassium, tremor, urinary retention, ocular effects, gallbladder disorders, intestinal obstruction, and anticholinergic effects. Pneumonia and LRTI were also assessed as AEs of special interest as these are common in the COPD patient population. Additional symptomatic endpoints included COPD exacerbations (incidence and time to first COPD exacerbation) and rescue medication use. Lung function endpoints included trough FEV_1_ and trough FVC.

For patients who did not need to re-screen as a result of a COPD exacerbation or LRTI during the run-in period (as outlined in the study design), there were a total of 7 study visits at: screening (Visit 1), randomization (Visit 2) and 1, 3, 6, 9, and 12 months (Visits 3–7). A follow-up assessment via telephone was conducted approximately 1 week after Visit 7, or following withdrawal. No subsequent active follow-up was performed. Spirometry assessments were conducted at Visit 1 (pre- and post-salbutamol) and pre-dose at Visits 2–7 to obtain FEV_1_ and FVC. ECGs and vital signs were assessed prior to salbutamol dosing for spirometry (Visit 1), and for Visits 2–7 immediately prior to dosing and at 10 and 45 minutes post dose. Holter ECG monitoring and the collection of clinical laboratory samples were conducted at Visit 1 and Visits 4–7.

### Sample size and statistical analyses

The sample size was determined based on ICH guidelines and practical considerations. To ensure that ≥300 patients completed the study (≥120 subjects per active treatment arm and ≥60 patients in the placebo arm), assuming a maximum withdrawal rate of 40% during the 52-week treatment period [16-20], it was planned that 500 patients would be randomized from approximately 50 study centers. The primary study population for all data presentation and analyses was the intent-to-treat (ITT) population, defined as all patients randomized to treatment who received at least one dose of study drug.

Formal statistical analyses were performed for vital signs and ECG parameters (analysis of covariance), time to first COPD exacerbation (Kaplan–Meier analysis, Cox proportional hazards model), trough FEV_1_ and trough FVC (repeated measures models). No formal statistical analyses were performed for other safety parameters or for comparison of active treatments with placebo. Results are presented as differences and confidence intervals (CIs). All other data are presented as patient numbers and percentages by study treatment group.

## Results

### Patients

Of the 563 patients randomized to treatment, one did not receive treatment and so 562 were included in the ITT population across 53 centers in the US (28% of patients), Romania (26%), Russian Federation (21%), South Africa (14%), Chile (7%), and Slovakia (4%). Of these, 342 patients completed the study (UMEC/VI 125/25 mcg, 63%; UMEC 125 mcg, 59%; placebo, 61%). Reasons for discontinuation included the meeting of protocol-defined stopping criteria (14%), AEs (9%), withdrawal of patient consent (7%), lack of efficacy (2%), protocol deviation (2%), study close/termination (2%), and loss to follow-up (2%). Although similar overall withdrawal rates were reported between UMEC/VI 125/25 mcg (37%), UMEC 125 mcg (41%), and placebo (39%), higher incidences of withdrawals due to protocol-defined stopping criteria were reported with UMEC/VI 125/25 mcg and UMEC 125 mcg (16% in each) compared with placebo (7%), particularly for ECG abnormalities (5–6% *vs* 0%) and Holter abnormalities (11–12% *vs* 7%). However, fewer patients were withdrawn due to a lack of efficacy on active treatments compared with placebo (≤1% *vs* 8%). For patients who reported ECG/protocol-defined stopping criteria as their primary reason for withdrawal, the ECG and Holter abnormalities meeting the withdrawal criteria are presented in Additional file 1: Tables S2 and S3; no single ECG or Holter abnormality was predominant.

Patient demographics, characteristics, and comorbid conditions were similar across treatment groups. Overall, patients had moderate-to-severe airflow obstruction, extensive smoking histories, and the majority were White and male (Table 1).

**Table 1:** Baseline demographics, characteristics and comorbid conditions

	**UMEC/VI**	**UMEC**	**Placebo**	**Total**
	**125/25 mcg**	**125 mcg**		
	(n = 226)*	(n = 227)*	(n = 109)*	(n = 562)*
**Age, years**				
Mean (SD)	61.4 (9.01)	61.7 (9.10)	60.1 (8.28)	61.3 (8.92)
**Sex, n (%)**				
Female	70 (31)	82 (36)	36 (33)	188 (33)
Male	156 (69)	145 (64)	73 (67)	374 (67)
**Ethnicity, n (%)**				
Hispanic/Latino	19 (8)	17 (7)	7 (6)	43 (8)
Not Hispanic/Latino	207 (92)	210 (93)	102 (94)	519 (92)
**Body mass index, kg/m**^**2**^				
Mean (SD)	27.89 (5.859)	28.05 (5.881)	27.65 (5.885)	27.91 (5.864)
**Smoking pack-years**∞				
Mean (SD)	43.7 (27.49)	39.2 (21.24)	42.8 (24.71)	41.7 (24.63)
**Pre-bronchodilator FEV**_**1 **_**(L)**	n = 225	n = 225	n = 108	n = 558
Mean (SD)	1.498 (0.5255)	1.432 (0.5120)	1.579 (0.5714)	1.487 (0.5311)
**Post-salbutamol% predicted FEV**_**1 **_**(L)**	n = 224	n = 225	n = 109	n = 558
Mean (SD)	55.0 (12.10)	54.2 (11.81)	55.1 (11.68)	54.7 (11.89)
**Reversibility to salbutamol, %**	n = 223	n = 224	n = 108	n = 555
Mean (SD)	12.7 (14.83)	14.2 (18.32)	11.9 (14.89)	13.1 (16.33)
**GOLD Stage, n (%)**	n = 224	n = 225	n = 109	n = 558
I (≥80% predicted FEV_1_)	0	0	1 (<1)	1 (<1)
II (≥50– <80% predicted FEV_1_)	137 (61)	129 (57)	71 (65)	337 (60)
III (≥30– <50% predicted FEV_1_)	87 (39)	96 (43)	37 (34)	220 (39)
IV (<30% predicted FEV_1_)	0	0	0	0
**Reversible to salbutamol, n (%)**^†^	n = 223	n = 224	n = 108	n = 555
Reversible	78 (35)	72 (32)	36 (33)	186 (34)
Non-reversible	145 (65)	152 (68)	72 (67)	369 (66)
**ICS use, n (%)**^‡^				
ICS users	80 (35)	73 (32)	40 (37)	193 (34)
ICS non-users	146 (65)	154 (68)	69 (63)	369 (66)
**Current medical conditions, n (%)**				
Any condition	190 (84)	196 (86)	88 (81)	474 (84)
Cardiovascular risk factors^§^	151 (67)	155 (68)	70 (64)	376 (67)
Cardiac disorders	74 (33)	80 (35)	37 (34)	191 (34)
Musculoskeletal and connective tissue disorders	84 (37)	64 (28)	32 (29)	180 (32)
Metabolism and nutrition disorders	35 (15)	35 (15)	18 (17)	88 (16)
Psychiatric disorders	33 (15)	36 (16)	15 (14)	84 (15)
Vascular disorders	26 (12)	26 (11)	15 (14)	67 (12)
Endocrine disorders	26 (12)	15 (7)	13 (12)	54 (10)
Nervous system disorders	19 (8)	19 (8)	11 (10)	49 (9)

*Unless otherwise specified; ∞Smoking pack-years = (number of cigarettes smoked per day/20) x number of years smoked prior to screening; ^†^reversible was an increase in FEV_1_ ≥12% and ≥200 mL following administration of salbutamol; non-reversible was an increase in FEV_1_ of <200 mL or a ≥200 mL increase that was <12% from pre-salbutamol FEV_1_; ^‡^ICS use was defined as those patients who were currently taking ICS medications at the Screening Visit; ^§^cardiovascular risk factors defined as current medical history of angina, myocardial infarction, stroke, diabetes, hypertension, or hyperlipidemia.

FEV_1_, forced expiratory volume in one second; GOLD, Global initiative for chronic Obstructive Lung Disease; ICS, inhaled corticosteroid; SD, standard deviation; UMEC, umeclidinium; VI, vilanterol.

Most patients had concurrent medical conditions in addition to COPD; the most commonly reported conditions were cardiovascular risk factors (67%; defined as a current medical history of angina, myocardial infarction, stroke, diabetes, hypertension, or hyperlipidemia) and cardiac disorders (34%) (Table 1).

In the 12 months prior to screening, 31% of patients in both active treatment groups and 36% of patients in the placebo group reported at least one COPD exacerbation that required oral/systemic corticosteroids and/or antibiotics. The proportion of patients who reported at least two of these exacerbations during this period was 11%, 14%, and 16% for UMEC 125 mcg, UMEC/VI 125/25 mcg, and placebo groups, respectively. For exacerbations that required hospitalization, 14%, 16%, and 17% of patients in the UMEC 125 mcg, UMEC/VI 125/25 mcg, and placebo groups reported at least one exacerbation, and 3%, 3%, and 6% reported at least two exacerbations.

### Outcomes

#### AEs

**Figure 2:**
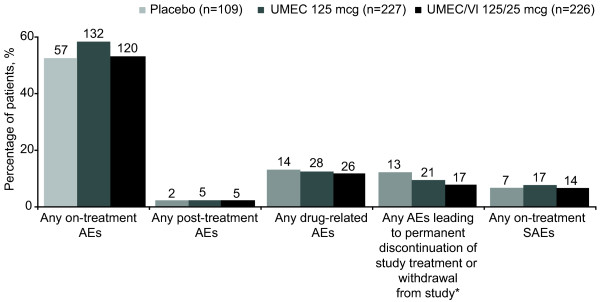
**AEs in the ITT population.** *Includes on-treatment and post-treatment AEs. AE, adverse event; ITT, intent-to-treat; SAE, serious AE; UMEC, umeclidinium; VI, vilanterol. Patient numbers are indicated above bars.

**Figure 3:**
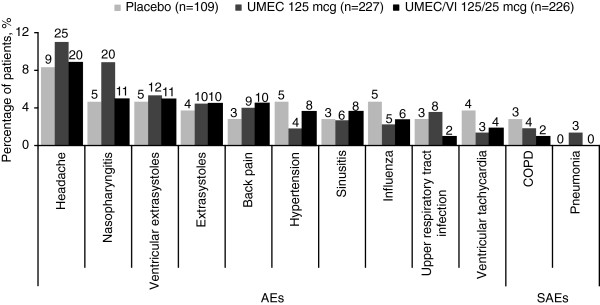
**On-treatment AEs and SAEs.** On-treatment AEs reported by ≥4% of patients in any treatment group and SAEs reported by ≥1% of patients in any treatment group in the ITT population. AE, adverse event; COPD, chronic obstructive pulmonary disease; ITT, intent-to-treat; SAE, serious AE; UMEC, umeclidinium; VI, vilanterol. Patient numbers are indicated above bars.

The incidence of on-treatment AEs, serious AEs (SAEs) and drug-related AEs was similar across active treatment groups and placebo (AEs: 52–58%; SAEs: 6–7%; drug-related AEs: 12–13%; Figures 2 and 3). On-treatment and post-treatment fatal AEs occurred at a low incidence across treatment groups (≤1%).Headache was the most common AE across all treatments (8–11%; Figure 3), followed by nasopharyngitis (5–9%) and ventricular extrasystoles (5% in each treatment group). In the UMEC/VI 125/25 mcg group, the incidence of the most common AEs (reported by ≥4% of patients) was similar to (≤1% difference) or less than placebo (Figure 3). In contrast, patients in the UMEC 125 mcg group reported incidences of headache and nasopharyngitis, ≥2% higher than placebo. AEs leading to permanent discontinuation or withdrawal were reported for 8% and 9% of patients in the UMEC/VI 125/25 mcg and UMEC 125 mcg groups, respectively, compared with 12% for placebo.The only on-treatment SAEs reported by ≥1% of patients in any treatment group (active or placebo) were COPD and pneumonia (all incidences ≤3%; Figure 3). Post-treatment and drug-related SAEs had a low incidence rate across treatment groups (≤1%).

In the pneumonia special interest group, a higher overall incidence of AEs was reported with UMEC 125 mcg (5%) compared with UMEC 125/25 mcg or placebo (both 2%) (Table 2). Patients receiving UMEC 125 mcg reported pneumonia (3%; half of whom had received ICS before screening and continued ICS treatment during the study), LRTI (1%), bronchitis (<1%), bronchitis viral (<1%), and pneumonitis (<1%); patients receiving UMEC/VI 125/25 mcg reported bronchitis, bronchitis viral, lobar pneumonia, LRTI, and sinobronchitis (all <1%); and patients receiving placebo reported bronchitis (2%) and tracheitis (<1%).

**Table 2:** Summary of on-treatment AEs of special interest

**Special interest AE group**	**Number (%) of patients**		
	**UMEC/VI**	**UMEC**	**Placebo**
	**125/25 mcg**	**125 mcg**	
	**(n = 226)**	**(n = 227)**	**(n = 109)**
Cardiovascular	34 (15)	49 (22)	25 (23)
Pneumonia	5 (2)	11 (5)	2 (2)
Anticholinergic syndrome	5 (2)	5 (2)	2 (2)
Effects on glucose	8 (4)	1 (<1)	0
Ocular effects	1 (<1)	1 (<1)	1 (<1)
Gallbladder disorders	0	2 (<1)	0
Effects on potassium	0	1 (<1)	0
Tremor	0	0	0
Urinary retention	0	0	0
Intestinal obstruction	0	0	0

AE, adverse event; UMEC, umeclidinium; VI, vilanterol.

In the glucose effect special interest group, a higher overall incidence of AEs was reported with UMEC/VI 125/25 mcg (4%) than UMEC 125 mcg (<1%) or placebo (0%) (Table 2). Two patients (<1%) in the UMEC/VI 125/25 mcg group who reported hyperglycemia also recorded diabetes mellitus as a current medical condition at screening. Overall, patients receiving UMEC/VI 125/25 mcg reported diabetes mellitus (1%), abnormal blood glucose (<1%), hyperglycemia (<1%), obesity (<1%), and weight decrease (<1%). Patients receiving UMEC 125 mcg reported hyperglycemia (<1%).

In the cardiovascular special interest group, a lower overall incidence of AEs was reported with UMEC 125/25 mcg (15%) than UMEC 125 mcg (22%) or placebo (23%; Table 2). The incidence of some individual cardiovascular events was ≥2% greater with UMEC 125 mcg than placebo: sinus tachycardia (UMEC 125 mcg, 3%; placebo, <1%); supraventricular extrasystoles (UMEC 125 mcg, 3%; placebo, <1%); supraventricular tachycardia (UMEC 125 mcg, 3%; placebo, <1%); and rhythm idioventricular (UMEC 125 mcg, 2%; placebo, 0%). Patients receiving UMEC/VI 125/25 mcg reported similar or lower incidences of these events (all <1%) than placebo.

No events were reported for any treatment in the special interest groups relating to tremor, urinary retention, or intestinal obstruction. Ocular effects and anticholinergic syndrome were reported in <1% and 2% of patients in each treatment group. Effects on potassium and gallbladder disorders were reported in <1% of patients in the UMEC 125 mcg treatment group, but in no patients receiving UMEC/VI 125/25 mcg or placebo (Table 2). Overall, no individual on-treatment AE in any of the special interest groups was reported by >5% of patients and incidences were generally similar across treatment groups.

#### Mortality

Five deaths occurred during the study: 4 (2%) in the UMEC 125 mcg group (spine metastases, liver metastases, pneumonia, and cardiac failure) and 1 (<1%) in the placebo group (coronary artery insufficiency). No deaths occurred in the UMEC/VI 125/25 group. None of the deaths were considered to be related to the study drug by the reporting investigator.

#### Clinical laboratory evaluations and vital signs

There was no clinically significant change from baseline in any clinical chemistry or hematology parameter in any treatment group, including glucose levels. Similarly, there was no evidence of a treatment-related effect on vital signs (systolic blood pressure, diastolic blood pressure, or pulse rate).

#### ECG parameters

The proportions of patients with one or more abnormal, clinically significant 12-lead ECG interpretation at any time post-baseline was similar across all treatment groups (23–26%). Post-baseline ECG abnormalities that occurred with an incidence ≥2% higher than placebo were frequent ventricular depolarization (UMEC/VI 125/25 mcg, 5%; UMEC 125 mcg, 6%; placebo, <1%), ectopic supraventricular beats (UMEC/VI 125/25 mcg, 3%; UMEC 125 mcg 4%; placebo, <1%), right bundle branch block (UMEC 125/25 mcg, 4%; UMEC 125 mcg, 3%; placebo, 2%), and first degree atrioventricular block (UMEC 125/25 mcg, 2%; UMEC 125 mcg, 3%; placebo <1%).

The proportions of patients with one or more abnormal, clinically significant Holter ECG interpretation at any time post-baseline was similar across all treatment groups (52–55%). Holter ECG recordings showed that the incidence of atrial arrhythmias with UMEC/VI 125/25 mcg was similar to placebo, but that some arrhythmias had a ≥2% greater incidence with UMEC 125 mcg compared with placebo; these included ectopic supraventricular beats, sustained supraventricular tachycardia and ectopic supraventricular rhythm.

The mean changes from baseline in heart rate were generally small in all treatment groups at all visits, with no evidence of a treatment-related effect. No clinically relevant treatment differences in QTc interval, PR interval, or heart rate were observed between treatment groups at any time point.

#### Trough FEV_1_ and FVC

**Figure 4:**
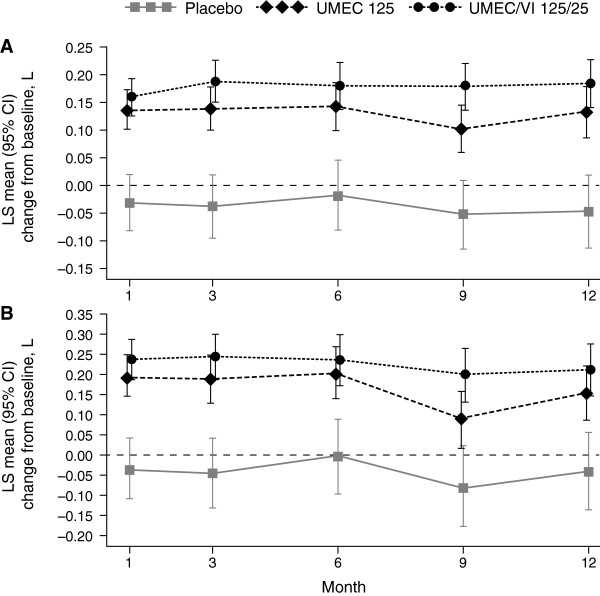
**LS mean change from baseline in trough FEV**_**1 **_**(A) and FVC (B).** CI, confidence interval; FEV_1_, forced expiratory volume in one second; FVC, forced vital capacity; LS, least squares; UMEC, umeclidinium; VI, vilanterol.

Greater mean changes from baseline in trough FEV_1_ and FVC were demonstrated for UMEC/VI 125/25 mcg and UMEC 125 mcg compared with placebo at all visits (Figure 4). At 12 months, UMEC/VI 125/25 mcg and UMEC 125 mcg had improved trough FEV_1_ in comparison with placebo by 0.231 L (95% CI: 0.153, 0.310) and 0.178 L (95% CI: 0.098, 0.258), respectively, and trough FVC by 0.252 L (95% CI: 0.135, 0.368) and 0.194 L (95% CI: 0.076, 0.312), respectively.

#### COPD exacerbations and rescue use

**Figure 5:**
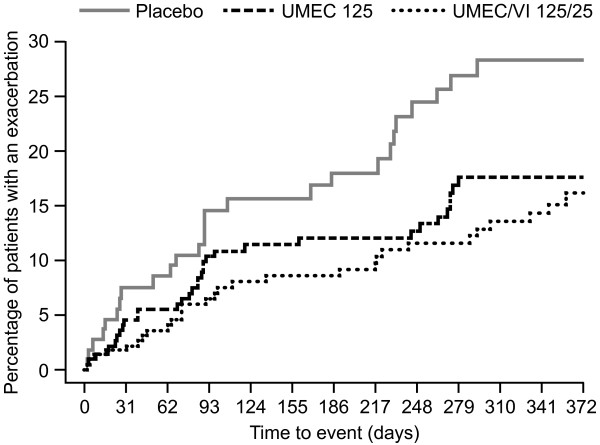
**Time to first COPD exacerbation.** COPD, chronic obstructive pulmonary disease; UMEC, umeclidinium; VI, vilanterol.

There were fewer patients reporting COPD exacerbations with UMEC/VI 125/25 mcg and UMEC 125 mcg (13% and 15%) compared with placebo (24%). COPD exacerbations resulting in hospitalization were also fewer with both UMEC/VI 125/25 mcg (6%) and UMEC 125 mcg (7%) compared with placebo (12%). Furthermore, based on analysis of time to first exacerbation, both UMEC/VI 125/25 mcg and UMEC 125 mcg were associated with a lower risk of COPD exacerbation compared with placebo (hazard ratio [HR] = 0.6, 95% CI: 0.3, 1.0, risk reduction 40%; HR = 0.4, 95% CI: 0.3, 0.8, risk reduction 60%, respectively) (Figure 5). On average, less rescue medication was required with UMEC/VI 125/25 mcg and UMEC 125 mcg (1.6 and 2.2 puffs/day) compared with placebo (2.6 puffs/day), while the mean change from baseline in the percentage of rescue-free days was greater with UMEC/VI 125/25 mcg (23%) than UMEC 125 mcg (13%) or placebo (11%).

## Discussion

This study aimed to examine the safety and tolerability of UMEC/VI 125/25 mcg and UMEC 125 mcg compared with placebo when administered over 12 months in patients with COPD. The population enrolled showed similar characteristics to the general COPD population and to that of previous clinical studies evaluating long-acting bronchodilators for the maintenance treatment of COPD [21-23]. The majority of patients had a history of cardiovascular risk factors (64–68%) at baseline, but the incidence was balanced across treatment groups. Overall, UMEC/VI 125/25 mcg and UMEC 125 mcg were well tolerated for up to 12 months, with no clinically meaningful treatment-related changes in vital signs or clinical laboratory parameters. No additive effects on AEs or safety assessments were noted with UMEC/VI 125/25 mcg compared with UMEC monotherapy, which is consistent with previous UMEC/VI studies [11,12,24].

Both AE and ECG data suggested that UMEC 125 mcg may be associated with an increase in atrial arrhythmias. However, the observations of supraventricular tachycardia and supraventricular extrasystoles were not associated with reports of clinically relevant symptoms such as hypotension or syncope, suggesting these arrhythmias may not be clinically meaningful. Nevertheless, data from other clinical trials suggests that atrial arrhythmias may be a class effect associated with anticholinergics [25]. Results from the Lung Health Study showed an increased risk of supraventricular tachycardia with the short-acting anticholinergic ipratropium bromide [26]. In the Understanding the Long-Term Impact of Tiotropium on Lung Function Trial (UPLIFT), there was an increased relative risk of tachyarrhythmias and atrial tachycardias reported as AEs for tiotropium compared with placebo [27], and in the tiotropium active comparator studies performed in the UMEC and UMEC/VI development program there were also some increases in atrial arrhythmias compared with baseline. A recently approved LAMA, aclidinium, has also been shown to have a greater incidence of non-sustained supraventricular tachycardias compared with placebo [28]. Interestingly, in this study the incidence of atrial arrhythmias with UMEC/VI 125/25 mcg was generally similar to placebo.

Although there were no formal efficacy endpoints in the present study, greater improvements from baseline in lung function were observed with both UMEC/VI 125/25 mcg and UMEC 125 mcg compared with placebo, together with reductions in COPD exacerbations and rescue medication use. These improvements were sustained over 12 months, indicating no tolerance issues and supporting findings from recent randomized controlled trials [11,12]. However, the present study was neither designed nor powered to detect differences in lung function outcomes or COPD exacerbations, and as such statistical conclusions cannot be drawn on the relative efficacy of the treatments.

## Conclusions

Overall, UMEC/VI 125/25 mcg and UMEC 125 mcg were well tolerated over 12 months of treatment in patients with COPD, and provided greater improvements from baseline in lung function and rescue medication use than placebo. These findings are supportive of the use of UMEC/VI and UMEC for the long-term treatment of COPD.

## Consent

All patients provided signed informed consent, prior to study participation, for the study results to be written up for medical journals.

## Abbreviations

AE: Adverse event; AV: Atrioventricular; CI: Confidence interval; COPD: Chronic obstructive pulmonary disease; ECG: Electrocardiogram; FEV_1_: Forced expiratory volume in one second; FVC: Forced vital capacity; HR: Hazard ratio; ICS: Inhaled corticosteroid; ITT: Intent-to-treat; GOLD: Global initiative for chronic Obstructive Lung Disease; LABA: Long-acting β_2_-adrenergic agonist; LAMA: Long-acting muscarinic antagonist; LRTI: Lower respiratory tract infection; LS: Least squares; SABA: Short-acting β_2_-adrenergic agonist; SAE: Serious adverse event; SD: Standard deviation; UMEC: Umeclidinium bromide; VI: Vilanterol.

## Competing interests

JFD is a consultant and advisor for Boehringer Ingelheim, Forest, GlaxoSmithKline, Mylan, Novartis, Sunovion, and is a consultant for PneumRx on the data safety monitoring board. DN has received fees from AstraZeneca, Boehringer Ingelheim, Forest Research, GlaxoSmithKline, Merck, and Novartis for serving on advisory boards or endpoint committees of clinical trials. JB, DO, and AC are employees of GlaxoSmithKline and hold stocks/shares in the company.

## Authors’ contributions

JFD and DN were involved in the conduct of the study, and the review and interpretation of the data, JB provided statistical input and analysis, and was involved in the review and interpretation of the data. DO and AC were involved in the planning and conduct of the study, and the review and interpretation of the data. All authors provided intellectual and critical input, contributed to the writing of the paper, and read and approved the final manuscript.

## Supplementary Material

Additional file 1: Table S1 Prohibited medications prior to Visit 1, by time interval. **Table S2.** Summary of ECG abnormalities meeting the withdrawal criteria. **Table S3.** Summary of Holter abnormalities meeting the withdrawal criteria. Click here for file
