# Supplementary material for: Safety and tolerability of once-daily umeclidinium/vilanterol 125/25 mcg and umeclidinium 125 mcg in patients with chronic obstructive pulmonary disease: results from a 52-week, randomized, double-blind, placebo-controlled study
Source: Respir Res. 2014 Jul 11;15(1):78. doi: 10.1186/1465-9921-15-78 (PMC4113670; doi:10.1186/1465-9921-15-78)
Supplement: Additional file 1: Table S1 — Prohibited medications prior to Visit 1, by time interval. Table S2. Summary of ECG abnormalities meeting the withdrawal criteria. Table S3. Summary of Holter abnormalities meeting the withdrawal criteria. [file 1465-9921-15-78-S1.doc]

**Additional file 1: Table S1. Prohibited medications prior to Visit 1, by time interval**

| **Medication** | **Time interval** |
| --- | --- |
| Depot corticosteroids | 12 weeks |
| Oral or parenteral corticosteroids | 6 weeks |
| Antibiotics for lower respiratory tract infection | 6 weeks |
| Cytochrome P450 3A4 strong inhibitors | 6 weeks |
| ICS/LABA combination products if ICS/LABA therapy is discontinued completely | 30 days |
| Use of ICS at a dose >1000 mcg/day fluticasone propionate or equivalent | 30 days |
| Initiation or discontinuation of ICS use | 30 days |
| Tiotropium | 14 days |
| Roflumilast | 14 days |
| Theophyllines | 48 hours |
| Oral leukotriene inhibitors (zafirlukast, montelukast, zileuton) | 48 hours |
| Oral β-agonists  Long-acting  Short-acting | 48 hours  12 hours |
| Inhaled LABAs | 48 hours |
| ICS/LABA combination products (only if discontinuing LABA therapy and switching to ICS monotherapy | 48 hours for the LABA component |
| Inhaled sodium cromoglycate or nedocromil sodium | 24 hours |
| Inhaled SABAs | 4 hours |
| Inhaled short-acting anticholinergics | 4 hours |
| Inhaled short-acting anticholinergic/SABA  combination products | 4 hours |
| Any other investigational medication | 30 days or within 5 drug half-lives (whichever is longer) |

ICS, inhaled corticosteroid; LABA, long-acting β-agonist; SABA, short-acting β-agonist.

**Additional file 1: Table S2. Summary of ECG abnormalities meeting the withdrawal criteria.**

**Legend: Summary of ECG abnormalities meeting the withdrawal criteria in patients who reported ECG protocol-defined stopping criteria as their primary reason for withdrawal (**ITT population).

|  | **Number (%) of patients** | | |
| --- | --- | --- | --- |
|  | **UMEC/VI 125/25 mcg (N=226)** | **UMEC 125 mcg (N=227)** | **Placebo  (N=109)** |
| **Patients withdrawn, n (%, ITT)** | 13 (6) | 12 (5) | 0 |
| Did not meet ECG stopping criteria, n (%, ITT) | 6 (3) | 3 (1) |  |
| Met ECG stopping criteria, n (%, ITT) | 7 (3) | 9 (4) |  |
| ECG abnormality, n (%, patients withdrawn) |  |  |  |
| Multifocal premature ventricular complexes | 3 (23) | 1 (8) | 0 |
| Sinus tachycardia ≥110 bpm | 2 (15) | 2 (17) | 0 |
| Bigeminy | 1 (8) | 2 (17) | 0 |
| Left bundle branch block | 0 | 2 (17) | 0 |
| Atrial fibrillation with rapid ventricular response  (rate >100 bpm) | 0 | 1 (8) | 0 |
| Bifascicular block | 0 | 1 (8) | 0 |
| Increase in heart rate ≥40 bpm relative to baseline | 0 | 1 (8) | 0 |
| Increase in QTcF >60 msec relative to baseline | 0 | 1 (8) | 0 |
| Junctional tachycardia (heart rate >100 bpm) | 1 (8) | 0 | 0 |
| Trigeminy | 0 | 1 (8) | 0 |

Abnormalities are only displayed if they were experienced by at least one subject; subjects may have more than one abnormality; denominators for abnormalities are the number of subjects with ECG protocol defined stopping criteria as the primary reason for withdrawal.

bpm, beats per minute; ECG, electrocardiogram; ITT, intent-to-treat; msec, millisecond; UMEC, umeclidinium bromide; VI, vilanterol.

**Additional file 1: Table S3. Summary of Holter abnormalities meeting the withdrawal criteria.**

**Legend: Summary of Holter abnormalities meeting the withdrawal criteria in patients who reported Holter protocol-defined stopping criteria as their primary reason for withdrawal (**ITT population).

|  | **Number (%) of subjects** | | |
| --- | --- | --- | --- |
|  | **UMEC/VI 125/25 mcg N=226** | **UMEC 125 mcg N=227** | **Placebo  N=109** |
| **Patients withdrawn (%, ITT)** | 26 (12) | 26 (11) | 8 (7) |
| Did not meet Holter stopping criteria, n (%, ITT) | 1 (<1) | 1 (<1) | 0 |
| Met Holter stopping criteria, n (%, ITT) | 25 (11) | 25 (11) | 8 (7) |
| Holter abnormality, n (%, patients withdrawn) |  |  |  |
| Non-sustained ventricular tachycardia  (>100 bpm, 3–30 beats) | 16 (62) | 9 (35) | 6 (75) |
| Sustained supraventricular tachycardia  (>100 bpm, >30 beats) | 4 (15) | 6 (23) | 0 |
| Premature ventriculation complex >4000 in 24-hour period | 5 (19) | 4 (15) | 0 |
| Idioventricular rhythm  (≤100 bpm, defined by wide QRS complex) | 2 (8) | 5 (19) | 1 (13) |
| Atrial fibrillation with rapid ventricular response (rate >100 bpm) | 1 (4) | 2 (8) | 0 |
| Second degree AV block (Mobitz type 2) | 0 | 0 | 1 (13) |
| Sinus tachycardia >120 bpm/hour for 4 consecutive hours | 0 | 1 (4) | 0 |

Abnormalities are only displayed if they were experienced by at least one subject; subjects may have more than one abnormality; denominators for abnormalities are the number of subjects with Holter protocol-defined stopping criteria as the primary reason for withdrawal.

AV, atrioventricular; bpm, beats per minute; ITT, intent-to-treat; UMEC, umeclidinium bromide; VI, vilanterol.
